# Screening and verification of hub genes in esophageal squamous cell carcinoma by integrated analysis

**DOI:** 10.1038/s41598-024-57320-7

**Published:** 2024-03-22

**Authors:** Hongqiang Wu, Peiyao Zhu, Peng Shu, Shuguang Zhang

**Affiliations:** Department of Thoracic Surgery, The First Hospital of China Medical University, No.155 North Nanjing Street, Shenyang, 110001 China

**Keywords:** ESCC, CDC6, GEO, Bioinformatics analysis, Cancer, Computational biology and bioinformatics

## Abstract

Esophageal squamous cell carcinoma (ESCC) is one of the most common malignant tumors. However, the mechanisms underlying ESCC tumorigenesis have not been fully elucidated. Thus, we aimed to determine the key genes involved in ESCC tumorigenesis. The following bioinformatics analyses were performed: identification of differentially expressed genes (DEGs); gene ontology and Kyoto Encyclopedia of Genes and Genomes pathway enrichment analysis; integrated analysis of the protein–protein interaction network and Gene Expression Profiling Interactive Analysis database for validation of hub genes. Finally, western blotting and qPCR were used to explore the expression of cell division cycle 6 (CDC6) in ESCC cell lines. Immunohistochemistry analysis of ESCC samples from patients and matched clinical characteristics was used to determine the effects of CDC6. A total of 494 DEGs were identified, and functional enrichment was mainly focused on cell cycle and DNA replication. Biological pathway analysis of the hub genes was closely related to the cell cycle. We found that CDC6 was upregulated in ESCC cell lines and patient tissues and was related to the clinicopathological characteristics of ESCC. In conclusion, this study identified hub genes and crucial biological pathways related to ESCC tumorigenesis and integrated analyses indicated that CDC6 may be a novel diagnostic and therapeutic target for ESCC.

## Introduction

Esophageal carcinoma (EC) is one of the most malignant tumors with a low 5-year survival rate and is the sixth leading cause of cancer death worldwide^1^. The two most common EC histological subtypes are esophageal adenocarcinoma (EAC) and esophageal squamous cell carcinoma (ESCC). ESCC accounts for 90% of all EC cases and is the predominant histological type in Eastern Asia^2^. New developments have been made in the multimodal treatment of several cancers; however, EC is still associated with a poor prognosis and high mortality rate. In most cases, EC is diagnosed at an advanced stage, disqualifying patients from surgical resection. Additionally, owing to chemotherapy resistance, EC recurrence is associated with poorer prognosis and shorter survival^3^. Therefore, identifying new biomarkers and targets for patients with ESCC is crucial.

The tumorigenesis of ESCC is related to genetic and environmental factors as well as individual lifestyle factors, including smoking and the consumption of hot drinks^4^. Gene mutations promote ESCC carcinogenesis via complex biological processes and multiple molecular interactions. For example, MTA1, SOX2, and FXYD-3 upregulation is associated with proliferation and angiogenesis in ESCC^5–7^. With the use of next-generation gene sequencing, novel genes and biomarkers have been identified^8^. Numerous differentially expressed genes (DEGs), critical for ESCC progression, have been reported^9–11^. However, the underlying mechanisms of ESCC tumorigenesis remain elusive and only a few hub genes are identified as ESCC biomarkers^12^.

Therefore, in this study, we aimed to identify DEGs and hub genes, and the underlying molecular mechanisms that contribute to ESCC progression through bioinformatics analyses of gene expression profiles obtained from four GEO databases.

## Results

### Identification of common DEGs

**Figure 1:**
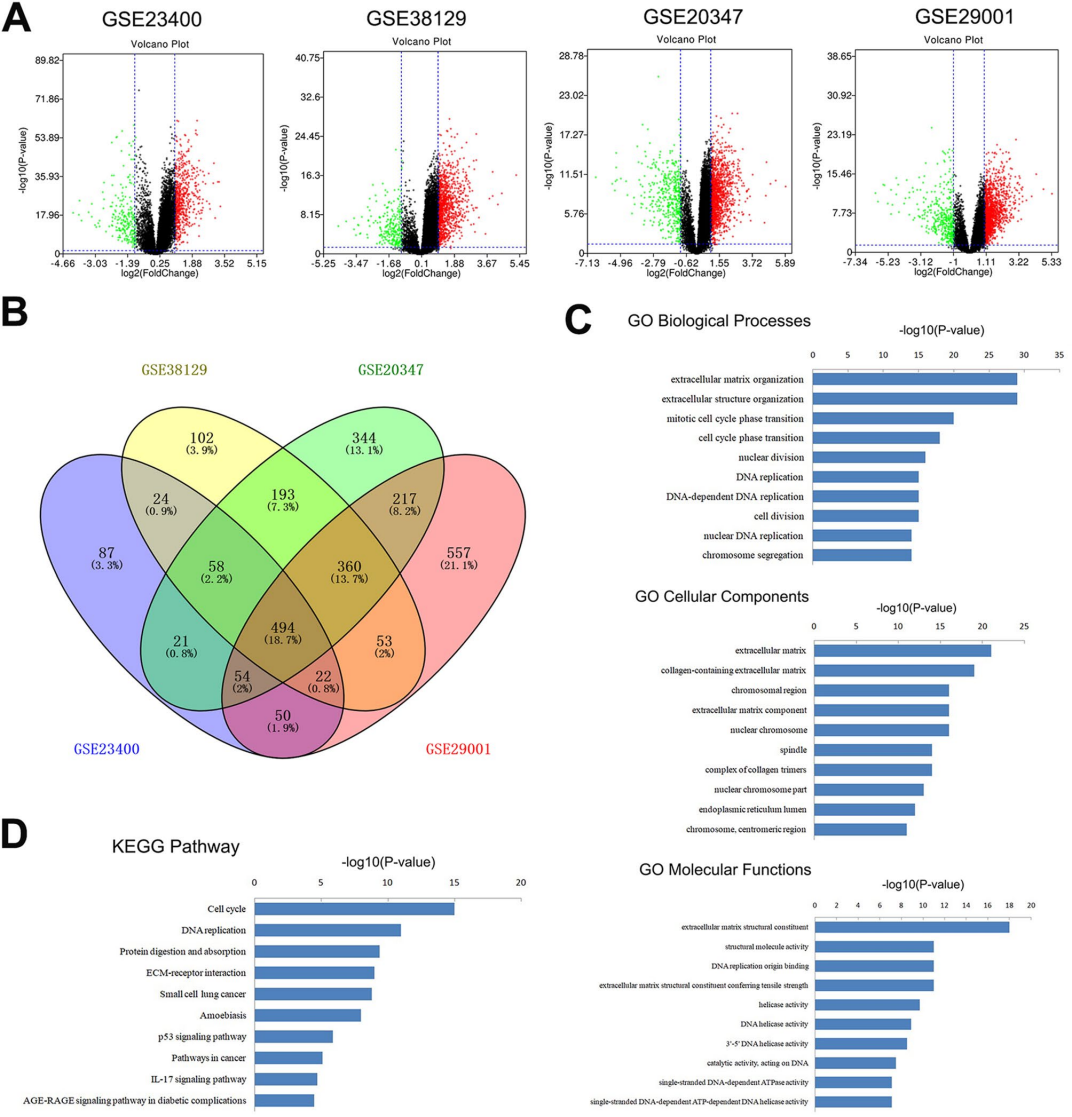
Identification of common DEGs, as well as GO and KEGG pathway enrichment analyses of identified DEGs. (**A**) Volcano plot of detectable genome-wide mRNA profiles in four independent GEO datasets, Red indicates DGEs with *p* < 0.05 and logFC (fold change) > 1. (**B**) Different color areas represented different datasets (blue oval represents GSE23400; yellow represents GSE38129; green represents GSE20347; pink represents GSE29001). Overlapping area in Venn diagrams represents overlapping DEGs among the four datasets. (**C**) GO analysis of DEGs in ESCC, including biological process, cell component, and molecular function. (**D**) KEGG pathway enrichment analysis of upregulated DEGs. DEGs, differentially expressed genes; GO, Gene Ontology; KEGG, Kyoto Encyclopedia of Genes and Genomes.

Gene expression data was obtained from four GEO datasets (GSE23400, GSE38129, GSE20347, and GSE29001). Using differential gene expression and Venn analyses, we identified 494 overlapping DEGs. Of the 494 genes, 348 were upregulated, and 146 were downregulated (Fig. 1A,B; Table 1).

**Table 1.** 348 upregulated and 146 downregulated DEGs were identified from the four GEO datasets of ESCC.

DEGs	Genes name
Upregulated (348)	ORC6, MYBL2, TTI1, RFC2, PDPN, ZNF200, CENPN, NEK2, RRS1, BID, SLC7A7, AURKA, CDCA3, COLGALT1, NCAPG, MTERF3, CENPM, HJURP, TROAP, ECT2, RBM28, LEPREL4, CAD, TTF2, FGD6, ACTL6A, APMAP, RPL39L, HOXD11, CDCA8, HSPBAP1, MELK, CBX3, CDK1, DLGAP5, POLE2, KIF14, ADSL, HOMER3, MMP11, KIF18B, TBCCD1, STIL, MAD2L1, GINS1, P4HA1, CHST12, RUVBL1, RFC4, FANCI, HSPE1, C5AR1, SAC3D1, GMNN, RAD54L, FCGR2A, RACGAP1, AURKB, OIP5, RAD54B, ATP2C1, ASAP1, TRMT12, MMP3, AUNIP, COL11A1, FSCN1, CCT6A, KIF11, TMEM185B, DBF4, ATAD2, ANP32E, IMPAD1, APOC1, SNAI2, DNAJC2, CEP55, COL1A1, DNA2, RAD51AP1, CDC45, MMP1, TOPBP1, ADAMDEC1, DTL, BIRC5, FAP, POPDC3, LACTB2, MCM10, ZNF146, MCM5, KIF2C, MEST, MCM6, TPX2, NCBP2, PFDN2, PARP1, E2F3, ASPM, FZD6, DPY19L4, SOAT1, SNX10, SLC38A6, MINPP1, LPCAT1, LAPTM4B, AHR, PLAU, PGAP1, SNRPG, CST4, RNASEH2A, F2R, RAI14, CCL18, SLC20A1, CKS1B, RCN1, RNF7, FJX1, HAT1, SOX4, TNFRSF12A, MLF1, POT1, ARPC1B, EFHD2, NCAPD2, CDK4, PRC1, YEATS2, DNMT1, HMGB3, DDX60, KIF20A, PCNA, HOXC10, KIF4A, PBK, SULF1, SLC16A1, TOP2A, TFRC, TMEM38B, GGH, SLC33A1, SERPINE1, NUP155, MCM4, LAMB3, INHBB, CALU, SERPINH1, VCAN, VOPP1, POLR2H, COL7A1, NUDT1, HPRT1, LSG1, PRKDC, MARCKSL1, GTF3C3, MCM2, CMC2, LOX, SLC25A32, COL3A1, FST, RPA1, CDC20, TTK, MAGEF1, CSGALNACT2, NUP107, CDC25B, ACVR1, U2SURP, NUSAP1, CDC6, PLAG1, MMP9, H2AFZ, CXCL1, NDC80, BGN, OLFML2B, MMP12, CCNE2, COL5A1, PANX1, CERS2, COL10A1, ADA, DDX39A, KREMEN2, NETO2, MCM7, FANCL, HSPD1, FXR1, NCF2, MFAP2, NREP, SNAPC1, ZWINT, RSAD2, SPP1, GINS2, PRKRIR, SMYD3, CDC7, MMD, DONSON, CTPS1, COL5A2, LMNB1, IGF2BP2, MSN, SP140L, JAG2, PTHLH, KIAA0101, HELLS, PLOD3, CKS2, TBL1XR1, SLC39A6, CCNB2, PCOLCE, COTL1, GINS4, DSG2, EZH2, TMEM97, CDH3, CCNB1, LUM, HSPH1, GALNT2, VRK1, USP18, TGIF1, CBS, MTHFD2, CXCL10, THY1, LY96, HEY1, TGFBI, SH3BP4, CXCL6, NID2, MAGEA12, ITGA6, TRIP13, ARMC1, PTPRK, SEL1L3, EPCAM, CCNB1IP1, EN1, COL1A2, HMGA2, PUS7, POSTN, FOXM1, AMIGO2, IFI44, IRS1, SSFA2, COL4A1, DFNA5, ATP1B3, LOC100129361, MFHAS1, PXDN, MMP10, UBE2C, MMP13, LAMB1, ISG15, ANKRD10, COL6A3, CCT2, TIMP1, RRM2, GPNMB, PRSS21, TUSC3, APOE, FAT1, ITGAV, APOL1, IFI6, BUB1B, CXCL8, SLC39A14, IFIT1, LAMA3, GCLM, TNC, ASPN, SPARC, FADD, LAMC2, PFN2, CDKN3, LYN, GALNT6, ODC1, ANO1, IGFBP3, CHST2, HLTF, PPFIA1, SLC6A8, KRT8, PSMB9, CYP24A1, PPAP2C, ALCAM, NELL2, ACKR3, TMEM45A, APOBEC3B, SLC7A11, MMP2, CALB1, COL4A2, IFI44L, KRT75, MAGEA6, BST2, MMP7, LGALS1, CXCL9, MAGEA11, RBP1, COL4A5, LAMP3, GREM1, CXCL11, NTS
Downregulated (146)	SCIN, PAIP2B, CAPN5, CYP4F12, CXCR2, GALNT12, CYP2J2, ACPP, MPC1, CWH43, COBL, UPK1A, ZNF426, ABLIM3, ABLIM1, UBL3, CPEB3, CEACAM1, FMO2, ID4, FLG, SASH1, GMDS, NUCB2, ENDOU, KAT2B, ADIRF, HPGD, EHD3, GPR110, MGLL, RRAD, SLC24A3, GPD1L, PPP1R3C, CRISP3, ECHDC2, TMPRSS2, BEX4, HLF, PDZD2, SLURP1, MAL, EPS8L1, GDPD3, STK39, IL1RN, SCEL, FUT3, CYP4B1, RORA, GYS2, CYP2C18, ADH1B, CLIC3, GPX3, BBOX1, EPB41L3, CES2, BLNK, TTC9, SULT2B1, TM7SF2, ANXA9, EPHX3, TRIP10, CCNG2, KLK13, CRNN, SPINK5, PPL, KANK1, SCNN1B, HSPB8, KLF4, TMOD3, C1orf116, CYP3A5, GLTP, EMP1, DHRS9, TGM3, FUT6, MXD1, CITED2, PITX1, RANBP9, LPIN1, PSCA, TMPRSS11E, ECM1, CEACAM6, OR7E14P, CLCA4, CRCT1, CRYAB, IL36A, TMPRSS11D, DUSP5, CRABP2, UPK3B, SERPINB13, SERPINB1, IL18, GABRP, IVL, HOPX, EHF, ZNF365, ALOX12, PRSS3, C2orf54, SYNPO2L, EPS8L2, NMU, RHCG, DHRS1, RAB11FIP1, ERO1L, OBFC1, CEACAM7, EVPL, KRT4, TGM1, SPRR2C, MALL, PLAC8, KRT13, ZNF185, CD24, SERPINB2, SPRR3, LYPD3, PTK6, AQP3, EREG, KLK12, AIM1, KRT24, CDA, KLK11, ABCA8, SPRR2B, PDZK1IP1, SLPI, LCN2

### Gene ontology (GO) and kyoto encyclopedia of genes and genomes (KEGG) pathway enrichment analyses of the common DEGs

We attempted to determine the roles of the identified DEGs in the progression of ESCC. Functional and pathway enrichment analyses were performed using Database for Annotation, Visualization, and Integrated Discovery (DAVID) software. GO analysis revealed that the biological processes of the DEGs (Supplementary Material 1) were significantly enriched in extracellular matrix organization, mitotic cell cycle phase transition, nuclear division, DNA replication, and chromosome segregation. Enriched cell components of the DEGs were primarily involved in the extracellular matrix, chromosomal region, spindle, nuclear chromosome, and endoplasmic reticulum lumen. Changes in molecular function were mainly enriched in extracellular matrix structural constituents, structural molecule activity, DNA replication origin binding, and DNA helicase activity (Fig. 1C). KEGG pathway analysis of the upregulated genes (Supplementary Material 2) revealed involvement in the cell cycle, DNA replication, protein digestion and absorption, extracellular matrix (ECM)–receptor interaction, small cell lung cancer, amoebiasis, p53 signaling pathway, cancer pathway, IL-17 signaling pathway, and AGE-RAGE signaling pathway in diabetic complications^13–16^(Fig. 1D).

### Protein–protein interaction (PPI) network and module analysis

**Figure 2:**
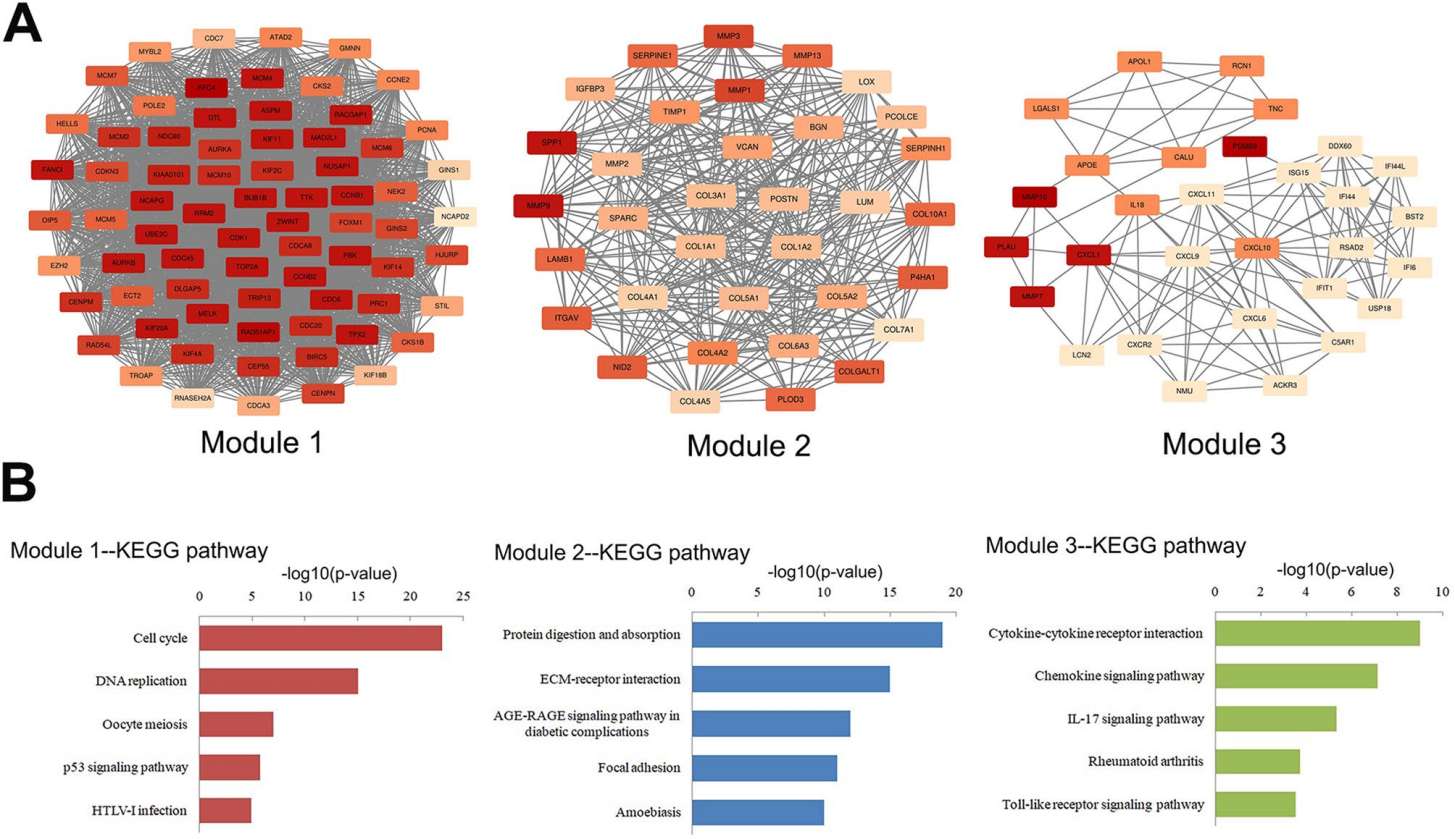
Significant modules and enrichment analyses of DEGs. (**A**) Top three modules from PPI network of DEGs. (**B**) Results of KEGG pathway analysis of DEGs in these three modules.

The PPI network of the DEGs was constructed using the STRING database and Cytoscape software. The top three significant modules (Supplementary Material 3) were selected using the MCODE plug-in in Cytoscape (Module 1: MCODE score = 66.056; Module 2: MCODE score = 21.758; Module 3: MCODE score = 8.621) (Fig. 2A). We then analyzed the functions of these modules. The enriched KEGG pathway showed that Module 1 consisted of 73 nodes and 2378 edges mainly enriched in the cell cycle, DNA replication, and oocyte meiosis. Module 2, containing 34 nodes and 359 edges, was primarily correlated with protein digestion and absorption by the ECM receptor. Module 3, which included 30 nodes and 125 edges, primarily correlated with cytokine–cytokine receptor interactions, chemokine signaling pathways, and IL-17 signaling pathways^13–16^ (Fig. 2B).

### Expression level of hub genes

**Figure 3:**
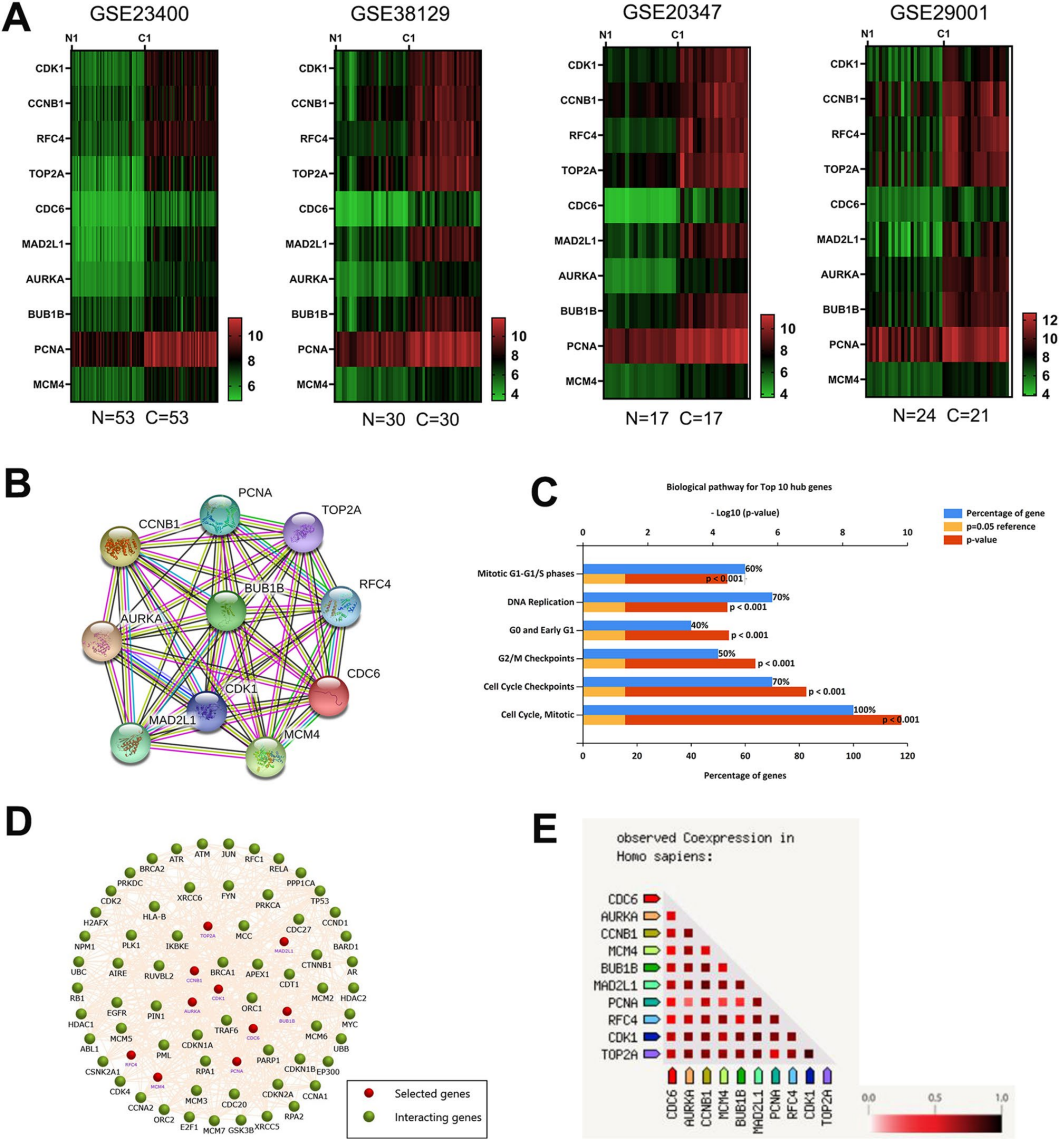
Comprehensive analysis of hub genes. (**A**) Hierarchical clustering heatmap of hub genes from four databases. (**B**). PPI network of the top 10 hub genes through the STRING database. (**C**) Biological pathway for the top 10 hub genes. (**D)**. Network of the top 10 hub genes and the other 50 frequently altered genes; red nodes represent hub genes and green nodes represent the co-expression genes. (**E**). Results of co-expression analysis of the top 10 hub genes.

Based on the degree of connectivity, the top 10 significant hub genes were filtered (Table 2). We created a heatmap of the 10 most essential hub genes, based on four data series (GSE23400, GSE38129, GSE20347, and GSE29001) and found that the hub genes could distinguish ESCC samples from normal samples (Fig. 3A). The PPI network of the 10 hub genes was predicted using the STRING database and constructed using Cytoscape (Fig. 3B). The biological pathways for the top 10 hub genes were cell cycle, mitosis, cell cycle checkpoints, G2/M checkpoints, G0 and Early G1, DNA replication, and Mitotic G1-G1-/S phases (Fig. 3C). Furthermore, we constructed a network of hub and co-expressed genes (Fig. 3D) and performed gene co-expression analysis of the hub genes using the STRING database and FunRich software (version 3.1.3; http://www.funrich.org/), which indicated that the hub genes may strongly interact with each other (Fig. 3E).

**Table 2:** Top ten hub genes identified by MCODE scores with Degree method.

Gene Symbol	Description	MCODE Score
CDK1	Cyclin-dependent kinase 1	104
CCNB1	Cyclin B1	97
RFC4	Replication factor C subunit 4	94
TOP2A	Topoisomerase (DNA) II alpha	94
CDC6	Cell division cycle 6	89
MAD2L1	Mitotic arrest deficient 2 like 1	89
AURKA	Aurora kinase A	89
BUB1B	BUB1 mitotic checkpoint serine/threonine kinase B	88
PCNA	Proliferating cell nuclear antigen	88
MCM4	Minichromosome maintenance complex component 4	87

### Expression verification of hub genes and genetic alteration

**Figure 4:**
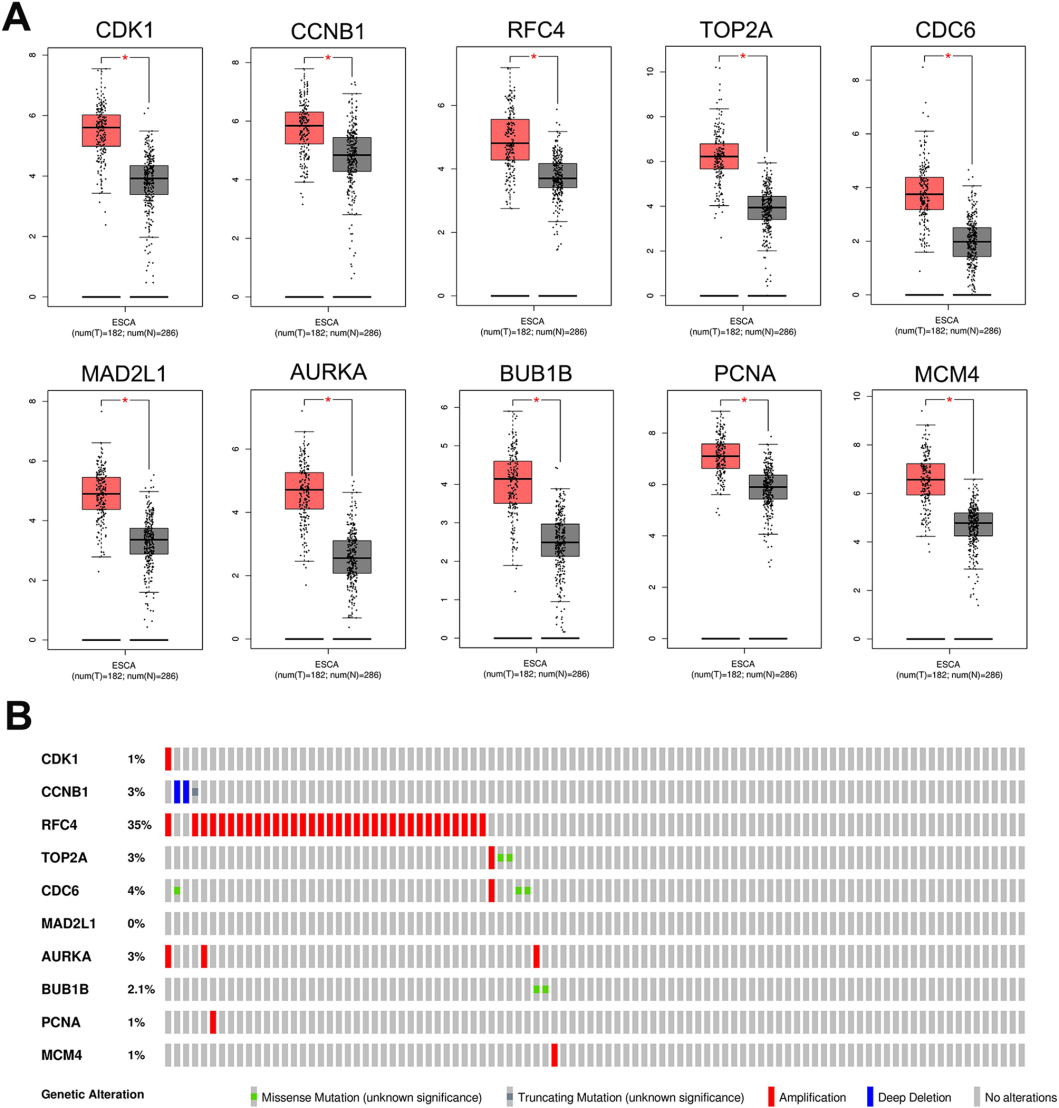
Validation of the top 10 gene expression levels based on the GEPIA. (**A**) Expression levels of top 10 hub genes in human ESCC. Gray and red boxes represent normal and tumor tissues, respectively. The results were consistent with the preceding consequence of this study. (**B**) Summary of genetic alternation of the top 10 hub genes in ESCC. *p < 0.05.

To investigate the transcriptional expression difference of the hub genes between tumor and normal tissues, we analyzed the datasets from the Gene Expression Profiling Interactive Analysis (GEPIA) database using one-way analysis of variance (ANOVA) (cut-off criteria: |log2FC|= 1 and *p* = 0.01). As shown in Fig. 4A, compared with normal tissues, the genes were significantly upregulated in tumor tissues. We then investigated the genetic alterations in the hub genes, and found that RFC4 and CDC6 had the top two frequencies of alteration (35% and 4%, respectively), including missense mutations, truncation mutations, amplifications, and deep deletions (Fig. 4B). These results suggest that RFC4 and CDC6 may play roles in the development and progression of ESCC and remained of great significance to further research.

### Receiver operating characteristic (ROC) analysis of hub genes and drug–hub gene interactions

**Figure 5:**
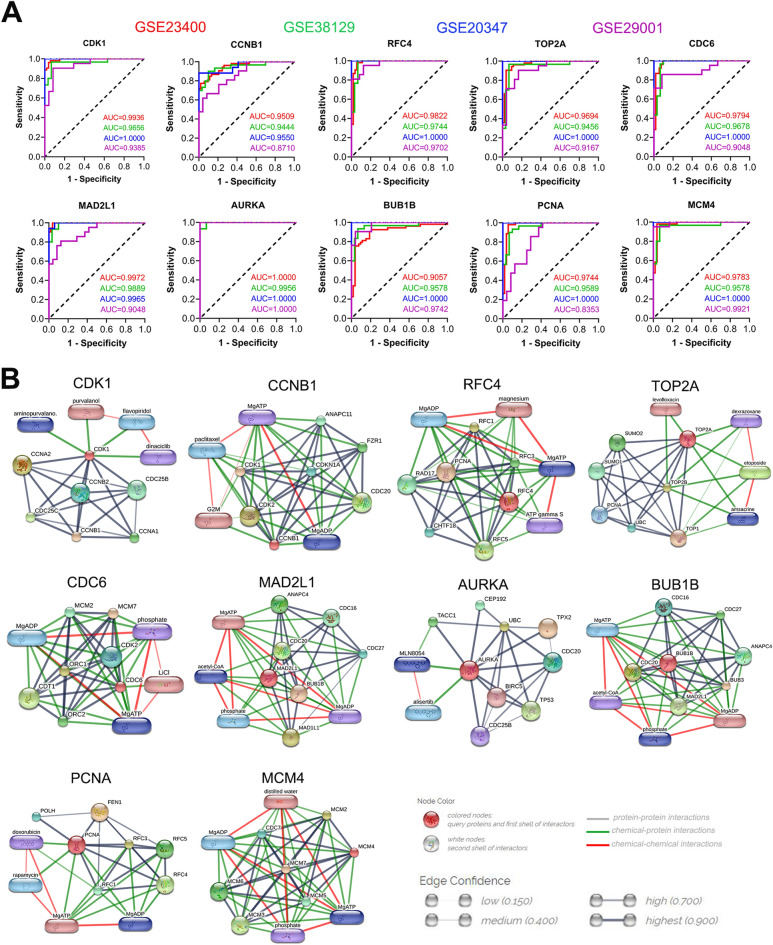
(**A**) ROC curves for ESCC diagnosis according to the AUC, red curve represents the GSE23400 dataset; green the GSE38129 dataset; blue the GSE20347 dataset; and purple the GSE29001 dataset. (**B**) Networks of drug–hub gene interactions. ROC, Receiver operator characteristic and AUC, area under the curve.

ROC curve analysis was performed to assess the diagnostic value of the top ten hub genes for ESCC (Fig. 5A). The expression levels of hub genes were extracted from four databases (GSE23400, GSE38129, GSE20347, and GSE29001). The area under the curve (AUC) of these genes ranged from 0.8353 to 1.000. Furthermore, we identified promising drugs associated with these hub genes. By searching druggable gene categories in DGIdb, we found that most hub genes matched with tumor suppressor genes (Table 3). Finally, 35 Food and Drug Administration (FDA) approved drugs (Table 4) targeting CDK1, TOP2A, AURKA, and PCNA were identified, with a large majority of the drugs inhibiting TOP2A. Furthermore, a downstream network of the 10 hub genes was constructed using the STITCH database (Fig. 5B). These modules showed that the hub genes were mainly associated with CDK, CDC, and MCM family members, and drugs including doxorubicin, rapamycin, etoposide, paclitaxel, amsacrine, levofloxacin, alisertib, MLN8054, aminopurvalano, dexrazoxane, MgATP, and MgADP.

**Table 3:** Druggable categories of hub genes of ESCC.

Druggable gene category	Matching gene count*	Matching gene(s)	Non-matching gene(s)
Tumor suppressor	8	CDK1, CCNB1, TOP2A, CDC6, MAD2L1, AURKA, BUB1B, PCNA	RFC4
Druggable genome	5	CDK1, CCNB1, TOP2A, AURKA, BUB1B	RFC4, CDC6, MAD2L1, PCNA
Kinase	5	CDK1, CCNB1, CDC6, AURKA, BUB1B	RFC4, TOP2A, MAD2L1, PCNA
Serine threonine kinase	5	CDK1, CCNB1, CDC6, AURKA, BUB1B	RFC4, TOP2A, MAD2L1, PCNA
DNA repair	3	CDK1, RFC4, PCNA	CCNB1, TOP2A, CDC6, MAD2L1, AURKA, BUB1B
Histone modification	3	CDK1, CCNB1, AURKA	RFC4, TOP2A, CDC6, MAD2L1, BUB1B, PCNA
Clinically actionable	2	TOP2A, AURKA	CDK1, CCNB1, RFC4, CDC6, MAD2L1, BUB1B, PCNA
Drug resistance	2	CDK1, CCNB1	RFC4, TOP2A, CDC6, MAD2L1, AURKA, BUB1B, PCNA

*MCM4 gene category was not included on DGIdb website.

**Table 4:** FDA approved drugs targeting hub genes of ESCC.

Hub gene	Drug	Interaction type	Score
CDK1	Eltrombopag	Agonist	1
CDK1	Romiplostim	Agonist	1
TOP2A	Mitoxantrone	Inhibitor	13
TOP2A	Teniposide	Inhibitor	12
TOP2A	Amsacrine	Inhibitor	12
TOP2A	Etoposide	Inhibitor	10
TOP2A	Podofilox	Inhibitor	9
TOP2A	Valrubicin	Inhibitor	6
TOP2A	Epirubicin	Inhibitor	6
TOP2A	Doxorubicin	Inhibitor	4
TOP2A	Enoxacin	Inhibitor	4
TOP2A	Daunorubicin	–	3
TOP2A	Vincristine	–	2
TOP2A	Norfloxacin	Inhibitor	2
TOP2A	Levofloxacin	Inhibitor	2
TOP2A	Ofloxacin	Inhibitor	2
TOP2A	Pefloxacin	Inhibitor	2
TOP2A	Dexrazoxane	–	2
TOP2A	Lomefloxacin	Inhibitor	2
TOP2A	Dactinomycin	–	2
TOP2A	Finafloxacin	Inhibitor	2
TOP2A	Idarubicin	–	2
TOP2A	Hydroquinone	–	2
TOP2A	Doxorubicin hydrochloride	Inhibitor	1
TOP2A	Etoposide phosphate	Inhibitor	1
TOP2A	Paclitaxel	–	1
TOP2A	Idarubicin hydrochloride	Inhibitor	1
TOP2A	Daunorubicin hydrochloride	Inhibitor	1
TOP2A	Mitoxantrone dihydrochloride	Inhibitor	1
TOP2A	Daunorubicin citrate	Inhibitor	1
AURKA	Paclitaxel	–	2
AURKA	Tamoxifen	–	2
AURKA	Fluorouracil	–	2
PCNA	Capsaicin	–	2
PCNA	Pentoxifylline	–	2

### High expression levels of CDC6 and its clinicopathologic characteristics in ESCC

**Figure 6:**
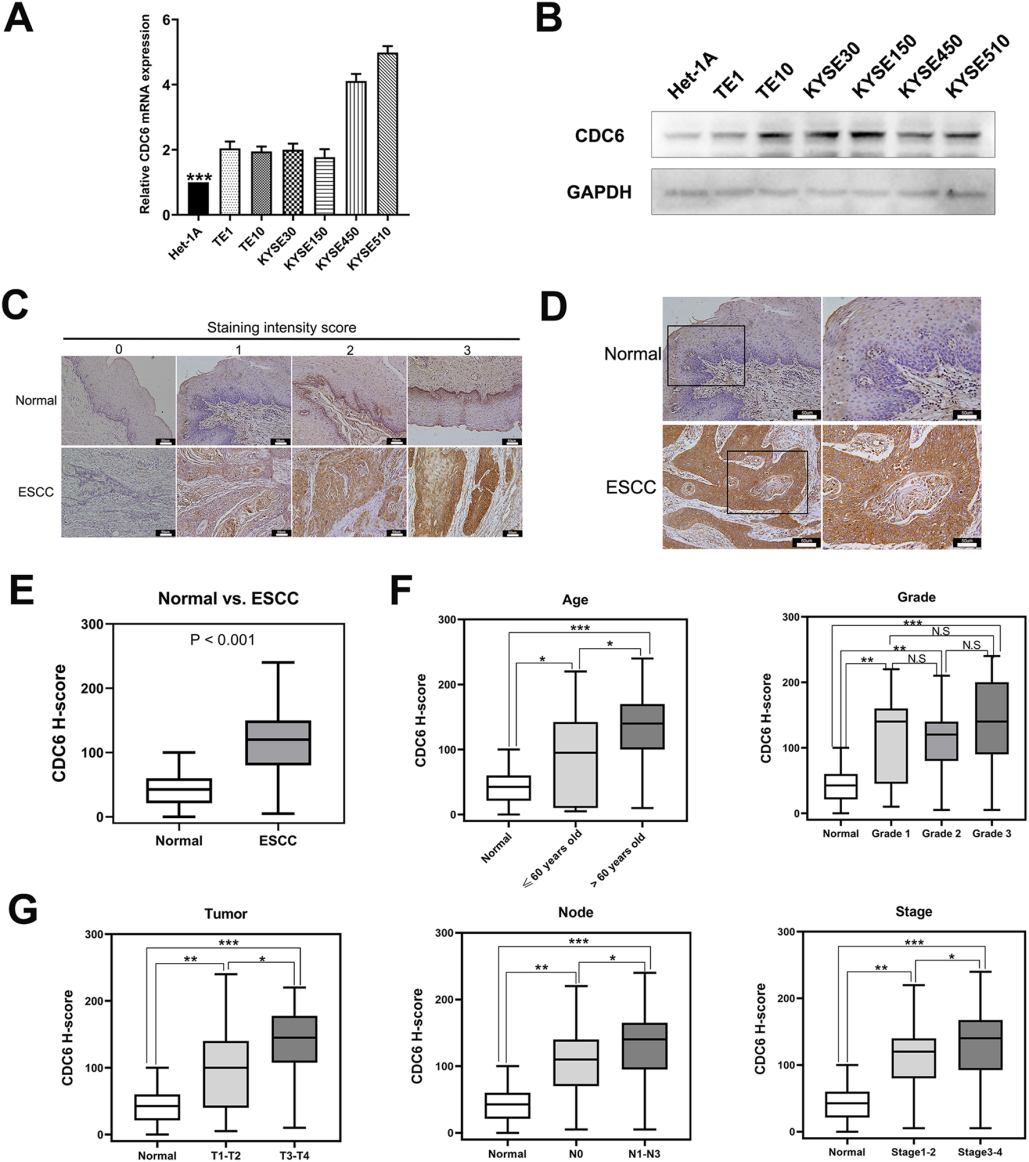
Verification of the CDC6 expression levels in ESCC and associated clinicopathologic characteristics. (**A**–**B**) mRNA and protein levels of CDC6 were detected in human esophageal epithelial cells (Het-1A) and ESCC cell lines using qPCR (**A**) and western blotting (**B**) (Het-1A vs. TE1, *p* < 0.0001; Het-1A vs. TE10, *p* = 0.0002; Het-1A vs. KYSE30, *p* < 0.0001; Het-1A vs. KYSE150, *p* = 0.0010; Het-1A vs. KYSE450, *p* < 0.0001; Het-1A vs. KYSE510, *p* < 0.0001). C. Immunohistochemical staining intensity scores of normal esophageal and tumor tissues ranging from 0 to 3 (× 200, 50µm). (**D**) Immunohistochemical staining for CDC6 expression in representative ESCC tissues and matched adjacent normal tissues (× 200, 50 µm). E. Compared to normal tissues, CDC6 was significantly upregulated in ESCC tissues. F–G. Correlation of the CDC6 H-score reflects the expression levels and clinicopathological characteristics, including age, grade, tumor size, lymph node metastasis, and stage. qPCR Quantitative polymerase chain reaction *p < 0.05, **p < 0.01, ***p < 0.001.

Through extensive literature review, we found that RFC4, with the highest mutation frequency, has been reported in ESCC, whereas few studies have focused on CDC6 in ESCC. To determine whether CDC6 plays a crucial role in ESCC, we evaluated the expression levels of CDC6 in ESCC cell lines. Both qPCR and western blotting showed that, compared to that in human esophageal epithelial cells, CDC6 was upregulated in ESCC cell lines (Fig. 6A,B). Furthermore, to detect the expression of CDC6 in ESCC samples, we performed immunohistochemical staining. Of the 35 samples, 26 exhibited high CDC6 expression levels, while the remaining samples showed low CDC6 expression (Fig. 6C,D). Statistical analysis of the H-score showed that, compared to normal esophageal tissues, CDC6 was upregulated in ESCC tissues (Fig. 6E). Therefore, we aimed to determine the relationship between CDC6 and the clinicopathological characteristics of ESCC using CDC6 H-scores (Supplementary Material 4). We selected five terms: age, grade, tumor size, lymph node metastasis, and stage. We found that the gene expression level of CDC6 was related to tumor size, age, lymph node metastasis, and stage, whereas it was not associated with tumor grade (Fig. 6F,G).

## Discussion

Although many studies have focused on ESCC tumorigenesis^17^, the incidence of ESCC remains high and its prognosis poor. Therefore, in this study, using expression data obtained from four datasets, we aimed to identify the critical genes and biological pathways involved in ESCC tumorgenesis. We identified 490 DEGs, and GO enrichment analysis showed that the identified DEGs were mainly enriched in the mitotic cell cycle phase transition, DNA replication origin binding, and biological pathways primarily enriched in the cell cycle, DNA replication, and protein digestion and absorption, indicating that the DEGs may be associated with the progression of ESCC.

We constructed a PPI network and selected the top three valuable modules. The biological pathway analyses of Module 1 revealed that the modules were mainly related to cell cycle and DNA replication, indicating that the genes may participate in the regulation of ESCC proliferation. Deregulation in proliferation remains is key to tumor development^18^. We found that part of Module 1 is related to the cell cycle and DNA replication, and previously, researchers have found that RFC4 is associated with increased DNA copy number alterations in ESCC^19^. CDK1, CCNB1, and CCNB2 are related to the cell cycle and are altered in ESCC^20^. Matrix metalloproteinases (MMPs) and collagen family members constitute the majority of Module 2. MMPs are involved in tumor invasion and metastasis. For instance, MMP13 participates in the proliferation and invasion of ESCC^21^. MMP9 can degrade Collagen IV in the basement membrane, which could promote invasion^22^. The ectopic expression of the collagen family is associated with the prognosis of some cancers^23–26^. The collagen family is associated with invasion and migration, which participate in epithelial to mesenchymal transition (EMT) and extracellular matrix remodeling^27^. Several genes of the collagen family have been identified as potential diagnostic and prognostic biomarkers for ESCC; however, the underlying molecular mechanisms need to be further expored^28,29^. The PPI network of Module 3 was mainly connected to the chemokine signaling pathway, cytokine–cytokine receptor interaction, and the IL-17 signaling pathway. Chemokine signaling pathways cause differences in the proliferation, angiogenesis, EMT, and metastasis of various cancers. CXCL_1_6/CXCR6 chemokine signaling mediates breast cancer^30^. IL-8 and CXCR-1 are involved in the EMT in colon carcinoma^31^. GROalpha-CXCR2 and GRObeta-CXCR2 signaling play crucial roles in ESCC cell proliferation^32^.

Based on the degree of connectivity, we considered the top 10 genes as hub genes. Both the heatmap and the results from the GEIPA database showed that the expression levels of the genes were significantly higher in tumor samples than in normal tissues, indicating that the genes may play key a role in the development of ESCC. The biological pathways of the 10 hub genes were mainly associated with cell cycle transition, indicating that the genes may affect the proliferation of tumor cells. Cyclin B1(CCNB1) silencing inhibited cell proliferation and facilitated senescence in pancreatic cancer^33^. CCNB1 is involved in the pathogenesis of esophagus carcinoma, and the CCNB1 upregulation is associated with a poor prognosis in patients with ESCC^34^. PCNA serves as a moving platform that allows DNA- and chromatin-interacting proteins to operate at the fork in a DNA sequence-independent manner, and targeting PCNA-1 can inhibit the proliferation of lung cancer cells^28,35,36^. Thus, the functions of the genes correspond to their respective biological pathways. Additionally, we constructed an interactive network including the 10 hub genes and the top 60 frequently altered neighboring genes in ESCC. Several significant genes, such as TP53, EGFR, and PARP1^37,38^ had direct or indirect connections to the 10 hub genes. These interactions indicate that hub genes may participate in ESCC tumorigenesis. The molecular mechanisms underlying these correlations warrant further study.

Using the hub genes, we searched for candidate drugs and 35 drugs with therapeutic efficacy against ESCC were identified. Four of the 10 hub genes, CDK1, TOP2A, AURKA, and PCNA, may be the potential drug targets. Notably, TOP2A is a promising target for ESCC^20^. In addition to doxorubicin, rapamycin, paclitaxel, and etoposide, we identified new drugs, such as levofloxacin, dexrazoxane, and amsacrine, that have not been used in ESCC. Further studies and clinical trials are required to explore their effects in ESCC. Most of the hub genes interacted with MgATP, MgADP, and phosphate. Intracellular ATP is critical for chemoresistance in colon cancer cells^39^ and the effect of drug–gene interactions on ESCC chemoresistance requires further research.

Alterations in hub genes included missense mutations, truncating mutations, amplification, and deep deletions. RFC4, with the highest mutation frequency, was upregulated in the early stage and was associated with early nodal metastases and tumor immunity in ESCC^19^. CDC6, with the second highest alteration frequency, has rarely been reported in ESCC. CDC6 forms part of the pre-replication complex that controls DNA replication licensing in the cell cycle^40^. Previous studies have shown that CDC6 was overexpressed in other tumors^41–43^. The expression level of CDC6 in ovarian cancer tissues was significantly higher than that in adjacent tissues and it was related to tumor stage, differentiation degree, lymph node metastasis, ascites and prognosis, which was an independent factor of ovarian cancer patients^44^. Mahadevappa et al. suggested that CDC6 expression was significantly increased in breast cancer tissues and correlated with poor prognosis of patients^45^. Further studies showed that down-regulation of CDC6 expression in bladder cancer could significantly inhibit a variety of malignant phenotypes of tumor cells^46^. After knocking out CDC6, the proliferation of tongue squamous cell carcinoma cells was inhibited^47^.

Additionally, CDC6 upregulation represses E-cadherin correlates with EMT^48^. CDC6 is also involved in the progression of ESCC induced by RFBP-and circular RNA circNELL2^49,50^. Consistent with the results from the bioinformatics analysis, CDC6 was upregulated in the ESCC cell lines. Furthermore, we explored the clinical significance of the findings by analyzing the CDC6 H-scores. The tumor samples had higher H-scores than normal samples. CDC6 expression was associated with tumor size, lymph node metastasis, and disease stage, indicating that CDC6 may promote the proliferation and invasion of tumor cells and serves as a novel ESCC therapeutic target. This study has some limitations. First, the number of clinical samples used to investigate the expression level of CDC6 was relatively small. Additionally, the effect of CDC6 on the prognosis of patients with ESCC remains unclear. The molecular mechanisms and biological effects of CDC6 in ESCC have not been fully elucidated.

## Conclusions

Using comprehensive bioinformatics analysis and in vitro assays, we demonstrated that the CDC6 gene plays a key role in the progression of ESCC and serves as a novel potential biomarker and therapeutic target for ESCC.

## Methods

### Data collection and DEGs extraction

Gene expression profiles of ESCC and adjacent normal tissues (GSE23400, GSE38129, GSE20347, and GSE29001) were downloaded from the GEO database (http://www.ncbi.nlm.nih.gov/geo/). The GSE23400 dataset consisted of 53 pairs of tumor tissues and matched noncancerous samples. GSE38129 consisted of 30 pairs of tumor and normal tissue samples. GSE20347 consisted of 17 pairs of tumor and normal esophageal tissue samples. GSE29001 consisted of 21 tumor samples and 24 normal tissues. DEGs were identified using R, and the significance procedures were as follows: the Affy package was used to perform background corrections and normalize the data, and then the Limma package was used to perform differential expression analysis. *p* < 0.05 and logFC (fold change) > 1 were set as cut-off criteria. Volcano plots for each dataset was drawn in R using the ggplot2 package, and the overlapping DEGs among the four microarrays were visualized using the Venn diagram tool (https://bioinfogp.cnb.csic.es/tools/venny/).

### Functional enrichment analyses

GO enrichment and KEGG pathway analyses were performed using the DAVID (available online: http://david.ncifcrf.gov). The GO results of crucial terms for cellular component (CC), biological process (BP), and molecular function (MF) were obtained by importing the DEGs into DAVID, and *p* < 0.01 was considered statistically significant.

### PPI network construction and analysis

PPI network comprising DEGs and hub genes were constructed using Metascape online analyses (https://metascape.org/gp/index.html#/main/step1), which could predict the interactions of proteins, and a combined score > 0.4 was considered statistically significant. Molecular interaction networks were constructed using Cytoscape (version 3.1.2; https://cytoscape.org/release_notes_3_2_1.html). The three most significant modules were identified by Molecular Complex Detection (MCOD, plug-in in Cytoscape software), which had MCODE scores > 3, false degree cut-off = 2, node score cut-off = 0.2, maximum depth = 100, and false k-score = 2. Functional enrichment analysis of each module was conducted using Metascape.

### Identification, analysis, and validation of hub genes

The top ten genes with the highest degrees of connectivity were selected as hub genes. The hub genes within the four ESCC databases were visualized using cluster heatmaps drawn using GraphPad Prism heatmap (version 6.0; http://uone-tech.cn/graphpad-prism.html). The biological pathway and co-expression analyses of the ten hub genes was conducted using STRING (https://string-db.org/). We validated the expression levels and connections of the ten hub genes in ESCC tissues and matched normal tissues using GEPIA (http://gepia.cancer-pku.cn/index.html), an online database for analyzing gene expression profiles of tumors. The genetic alterations of the ten selected hub genes in ESCC were determined using cancer genomics prolifers obtained from the cBioCancer Genomic Portal (http://www.cbioportal.org/), which contains a large number of cancer genomics datasets.

### Analysis of drug–hub gene interactions

The 10 hub genes served as the promising targets for searching for candidate drugs through the DGIdb (http://dgidb.genome.wustl.edu/), drugs from more than one database, or PubMed references that are approved by the FDA. The target network of the hub genes was constructed using STITCH (http://stitch.embl.de/).

### Cell culture

Het-1A, TE-1, TE-10, KYSE30, KYSE150, KYSE450, and KYSE510 cells lines were purchased from the Type Culture Collection of the Chinese Academy of Science (Shanghai, China), and were cultured in a RPMI-1640 medium supplemented with 10% fetal bovine serum (FBS, Biological Industries, Israel), 1% penicillin, and 1% streptomycin (Biological Industries, Israel) at 37 °C and 5% CO_2_.

### RNA extraction and quantitative PCR

RNA was extracted from the cultured cells using the miRNeasy Mini Kit (QIAGEN, Germany), quantified using a spectrophotometer (BioTek, USA) and synthesized into complementary DNA using the GoScript Reverse Transcription Kit (Promega, USA), according to the manufacturer’s instructions. Quantitative PCR was performed using a 7900HT qPCR System (Applied Biosystems, USA) and GoTaq qPCR System Kit (Promega, USA), according to the manufacturer’s instructions. The primer sequences used were as follows: CDC6: sense primer: 5′-GCACAGGCTACAATCAGT-3′, anti-sense: 5′-CGAGGAGAACAGGTTACG-3′; GAPDH: sense primer: 5′-TCTCTGCTCCTCCTGTTC-3′, anti-sense: 5′-GTTGACTCCGACCTTCAC-3′. The relative mRNA expression of CDC6 was calculated using the 2^−ΔΔCT^ method, normalized to that of endogenous GAPDH.

### Western blot analysis

Cells were lysed using a RIPA lysis buffer (Beyotime, China), supplemented with 1 mM phenylmethylsulfonyl fluoride (Beyotime, China) and 1 mM phosphatase inhibitor (MCE, USA). Protein concentrations were quantified using an Enhanced BCA Protein Assay Kit (Beyotime,China), according to the manufacturer’s instructions. Protein samples were added to SDS loading buffer and boiled at 95 °C for 10 min. The denatured protein samples were electrophoresed on a 10% SDS-PAGE gel (Biosharp, China) and transferred onto a polyvinylidene fluoride (PVDF) membrane (Merck Millipore, Germany) using the wet transfer method (we cut the SDS-PAGE gels according to the position of target protein before the protein samples were transferred onto PVDF membranes). CDC6 is 63 kDa, while GAPDH is 36 kDa. The final PVDF membranes contained all these components. The membrane was blocked with 5% skim milk (Solarbio, China) at room temperature for 2 h. Next, the membranes were incubated with anti-CDC6 antibody (Abcam, USA) and anti-GAPDH antibody (Abcam, USA) at 4 °C overnight with gentle shaking. The membranes were washed with tris-buffered saline and Tween 20 (TBST) solution and then incubated with a horseradish peroxidase-conjugated goat anti-rabbit secondary antibody (Thermo Fisher Scientific, USA) at room temperature for 2 h. Protein bands were visualized using enhanced chemiluminescence (ECL) detection reagent (Beyotime, China). GAPDH was used as the internal control. All experiments were performed in triplicate. Densitometry analysis for western blots was conducted using ImageJ software (version 1.8.0, https://downloads.digitaltrends.com/imagej/windows).

### Immunohistochemistry (IHC)

The Ultra Sensitive SP (Mouse/Rabbit) IHC Kit (Maixin, China), primary antibodies against CDC6 (Abcam, USA), and horseradish peroxidase-conjugated goat anti-rabbit secondary antibody (Abcam, USA) were used for IHC analysis. ADAB (3, 3′-Diaminobenzidine) kit (Maixin, China), which produces brown reactions, was used, according to the manufacturer’s instructions, for staining. The slides were counterstained with hematoxylin, dehydrated, made transparent, and mounted with neutral balsam. The IHC scores were independently assessed by two experienced pathologists who were blinded to any other information. Scores were based on the average staining intensity and staining ratio in five random high-power fields of view. Staining intensity scores were 0 (no staining), 1 (weak staining), 2 (moderate staining), and 3 (strong staining). We defined the staining ratio of positive cells as follows: 0 (< 5%), 1 (5–25%), 2 (26–50%), 3 (51–75%), and 4 (> 75%). The final IHC score ranged from 0 to 12 and was calculated as the staining intensity multiplied by the staining ratio. A final score ≥ 4 indicated high CDC6 expression, and < 4 indicated low CDC6 expression. Informed consent was obtained from all patients.

### Statistical analysis

Data were analyzed using Statistical Package for the Social Sciences (SPSS, version 20) (https://spss.en.softonic.com/). Diagrams and ROC curve analyses were generated using GraphPad Prism software (version 6.0, San Diego, CA, USA). Dunnett's multiple comparison test was used to compare the CDC6 mRNA levels between ESCC and normal esophageal epithelial cell lines. All values are represented as mean ± standard deviation. The relationship between CDC6 and clinical factors was analyzed using χ2 independence or Fisher’s exact tests. Statistical significance was set at *p* < 0.05.

### Informed consent

Tumor samples and clinical data were collected and used with the guidance of the Declaration of Helsinki and approved by the Ethics committee of the First Hospital of China Medical University.

Written informed consent has been obtained from the patients to publish this paper.

### Supplementary Information

Supplementary Information 1.

Supplementary Information 2.

## Data Availability

The data used to support the findings of this study are available from the corresponding author upon request.
