# Supplementary figures and images for: Screening and verification of hub genes in esophageal squamous cell carcinoma by integrated analysis
Source: Sci Rep. 2024 Mar 22;14:6894. doi: 10.1038/s41598-024-57320-7 (PMC10959922; doi:10.1038/s41598-024-57320-7)

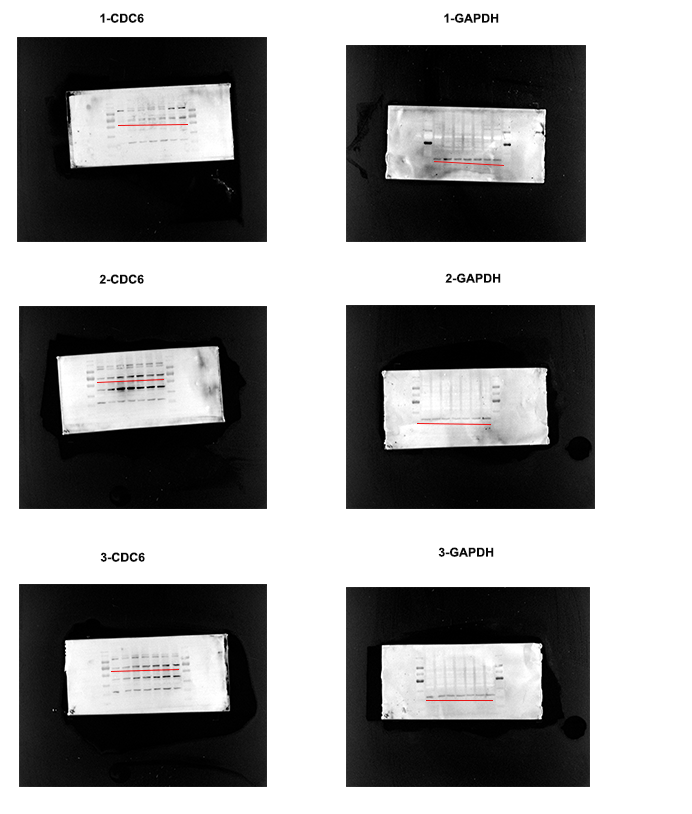

Supplement: Supplementary file 2 — Supplementary Information 2. [file 41598_2024_57320_MOESM2_ESM.tif]
